# Multi-omics data integration for enhanced cancer subtyping via interactive multi-kernel learning

**DOI:** 10.1093/bib/bbaf687

**Published:** 2025-12-17

**Authors:** Hongyan Cao, Tong Wang, Zhaoyang Xu, Xin Zhao, Gaiqin Liu, Xiaoling Yang, Ruiling Fang, Yanhong Luo, Ping Zeng, Hongmei Yu, Yanbo Zhang, Yuehua Cui

**Affiliations:** Department of Health Statistics, Shanxi Provincial Key Laboratory of Major Diseases Risk Assessment, School of Public Health, Shanxi Medical University, No. 56 South Xinjian Road, Yingze District, Taiyuan, Shanxi 030001, PR China; MOE Key Laboratory of Coal Environmental Pathogenicity and Prevention, Shanxi Medical University, No. 56 South Xinjian Road, Yingze District, Taiyuan, Shanxi 030001, PR China; Department of Health Statistics, Shanxi Provincial Key Laboratory of Major Diseases Risk Assessment, School of Public Health, Shanxi Medical University, No. 56 South Xinjian Road, Yingze District, Taiyuan, Shanxi 030001, PR China; Academy of Medical Sciences, Shanxi Medical University, No. 56 South Xinjian Road, Yingze District, Taiyuan, Shanxi 030001, PR China; Department of Health Statistics, Shanxi Provincial Key Laboratory of Major Diseases Risk Assessment, School of Public Health, Shanxi Medical University, No. 56 South Xinjian Road, Yingze District, Taiyuan, Shanxi 030001, PR China; Academy of Medical Sciences, Shanxi Medical University, No. 56 South Xinjian Road, Yingze District, Taiyuan, Shanxi 030001, PR China; Department of Health Statistics, Shanxi Provincial Key Laboratory of Major Diseases Risk Assessment, School of Public Health, Shanxi Medical University, No. 56 South Xinjian Road, Yingze District, Taiyuan, Shanxi 030001, PR China; Department of Health Statistics, Shanxi Provincial Key Laboratory of Major Diseases Risk Assessment, School of Public Health, Shanxi Medical University, No. 56 South Xinjian Road, Yingze District, Taiyuan, Shanxi 030001, PR China; MOE Key Laboratory of Coal Environmental Pathogenicity and Prevention, Shanxi Medical University, No. 56 South Xinjian Road, Yingze District, Taiyuan, Shanxi 030001, PR China; Department of Thoracic Oncology, Shanxi Bethune Hospital, Shanxi Academy of Medical Sciences, Tongji Shanxi Hospital, Third Hospital of Shanxi Medical University, No. 99 Longcheng Street, Xiaodian District, Taiyuan, Shanxi 030032, PR China; Department of Health Statistics, Shanxi Provincial Key Laboratory of Major Diseases Risk Assessment, School of Public Health, Shanxi Medical University, No. 56 South Xinjian Road, Yingze District, Taiyuan, Shanxi 030001, PR China; MOE Key Laboratory of Coal Environmental Pathogenicity and Prevention, Shanxi Medical University, No. 56 South Xinjian Road, Yingze District, Taiyuan, Shanxi 030001, PR China; Department of Health Statistics, Shanxi Provincial Key Laboratory of Major Diseases Risk Assessment, School of Public Health, Shanxi Medical University, No. 56 South Xinjian Road, Yingze District, Taiyuan, Shanxi 030001, PR China; MOE Key Laboratory of Coal Environmental Pathogenicity and Prevention, Shanxi Medical University, No. 56 South Xinjian Road, Yingze District, Taiyuan, Shanxi 030001, PR China; Department of Biostatistics, School of Public Health, Xuzhou Medical University, No. 209 Tongshan Road, Yunlong District, Xuzhou, Jiangsu 221004, PR China; Department of Health Statistics, Shanxi Provincial Key Laboratory of Major Diseases Risk Assessment, School of Public Health, Shanxi Medical University, No. 56 South Xinjian Road, Yingze District, Taiyuan, Shanxi 030001, PR China; MOE Key Laboratory of Coal Environmental Pathogenicity and Prevention, Shanxi Medical University, No. 56 South Xinjian Road, Yingze District, Taiyuan, Shanxi 030001, PR China; Department of Health Statistics, Shanxi Provincial Key Laboratory of Major Diseases Risk Assessment, School of Public Health, Shanxi Medical University, No. 56 South Xinjian Road, Yingze District, Taiyuan, Shanxi 030001, PR China; MOE Key Laboratory of Coal Environmental Pathogenicity and Prevention, Shanxi Medical University, No. 56 South Xinjian Road, Yingze District, Taiyuan, Shanxi 030001, PR China; Department of Statistics and Probability, Michigan State University, 619 Red Cedar Road, East Lansing, MI 48824, USA

**Keywords:** interactive multi-kernel learning, multi-omics data integration, omics-omics interaction, subtype identification, unsupervised multi-kernel learning

## Abstract

Cancer is a highly heterogeneous disease characterized by complex molecular changes. Subtypes identified through multi-omics data hold significant promise for improving prognosis and facilitating personalized precision treatment. Recent multi-omics integration methods have mostly focused on capturing complementary information from different data types, often overlooking potential interactions between omics data. Here we develop a novel method named interactive multi-kernel learning (iMKL), which incorporates omics-omics interactions alongside heterogeneous data types under the unsupervised multi-kernel learning framework, to improve subtype identification. Using the sample-similarity kernel for each dataset, we propose a joint Hadamard product strategy to capture higher-order interactive effects from different omics data types. We applied iMKL to two renal cell carcinoma (RCC) datasets*—*clear renal cell carcinoma (ccRCC) and type II papillary renal cell carcinoma (type II pRCC)—both including miRNA expression, mRNA expression, and DNA methylation data. Stability analysis through random sampling of patients or features demonstrated that iMKL exhibits strong robustness and accuracy in identifying patient subtypes. The identified subtypes revealed dramatic differences in patient survival, with both ccRCC and type II pRCC classified into three distinct subtypes. The findings in the real application highlight potential biomarkers associated with adverse patient outcomes and demonstrate substantial advancement in cancer subtype identification. The iMKL method effectively identifies tumor molecular subtypes that are strongly associated with clinical features and survival rates, providing valuable insights for accurate cancer subtyping, clinical decision-making, and the realization of personalized treatment strategies.

## Graphical Abstract

**Graphical Abstract:**
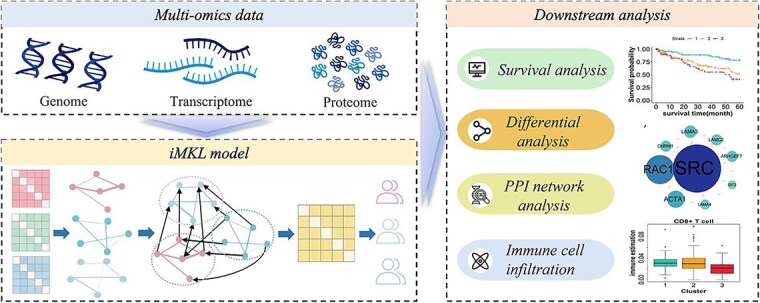

## Introduction

Cancers are highly heterogeneous diseases often with poor clinical prognoses. Patients with the same tumor type and histopathological characteristics frequently exhibit markedly diverse genomic landscapes [1]. These variations can significantly influence responses to diverse treatment modalities and have a significant impact on prognosis. Therefore, delineating cancer subtypes by discerning shared molecular features and correlating them with clinical outcomes is essential for improving prognosis and facilitating personalized precision treatment.

Remarkable advances in high-throughput technologies have facilitated the generation of exponentially growing, diverse multi-omics data, offering unprecedented opportunities for the improved stratification of cancer patients [2]. The integration of multi-omics data is widely recognized as a potential and valuable strategy for harnessing diverse genomic data types to investigate the pathogenesis and molecular subtyping of cancers [3]. A wide range of multi-omics integration methods have been developed, which can be classified into four distinct categories [4–6]. Early integration methods, such as LRAcluster [7], combine all datasets into a single large input matrix on which a single-omic clustering algorithm is applied. However, this approach assumes a homogeneous contribution of each data type, which can result in information loss and bias. Additionally, it increases data dimensionality, introducing challenges that surpass those faced with single-omic datasets [8]. In contrast, late integration methods, such as PINSPlus [9], cluster each dataset separately using single-omic algorithms, and the resulting clusters are subsequently integrated to obtain a comprehensive clustering solution. Nevertheless, this approach may result in the loss of weak signals present in individual single-omic datasets. The third category is statistical modeling-based methods (e.g. iCluster [10]). These methods assume a probabilistic distribution, powerful but run slower and are sensitive to feature selections. The fourth category, known as transformation-based methods (e.g. SNF [11]), integrates multi-omics data by transforming each dataset into an intermediate form through individual transformations (e.g. graph or kernel matrix), and subsequently integrating them into joint transformations for analysis [12]. These methods leverage the “kernel trick” for each dataset, allowing pairwise similarities between samples to be computed in a high-dimensional space. A prominent method in this category is unsupervised multiple kernel learning (UMKL) [13], which combines multiple kernels to form a unified meta-kernel within an unsupervised framework. This approach flexibly incorporates heterogeneous multi-omics data and assigns different weights to the kernels based on their relative contributions [14].

Among the four categories of multi-omics data integration methods, most focus on capturing shared (common) or complementary (distinct) information from different data types, while typically overlooking potential contributions of omics-omics interactions, i.e. interactions between distinct omics layers. The molecular regulatory mechanisms of cancer are highly complex, involving large-scale interactions among genomic factors such as miRNA, mRNA, and DNA methylation. These factors interact at multiple levels, forming interdependent and intricate networks [15–17] that cooperatively influence and regulate various biological behaviors in cancer [18]. Incorporating interaction effects within and across multi-omics data types can effectively enhance cancer subtyping performance. This necessitates the development of innovative computational methods tailored for multi-omics data integration. While the UMKL methods can capture complementary information embedded in each data type by combing all kernels to model sample similarity, they are not designed to borrow rich information due to omics-omics interactions. To alleviate this disadvantage, it is essential to incorporate omics-omics interaction information under the UMKL framework to achieve efficient and precise subtyping.

Recently, a number of kernel-based interaction detection algorithms have been developed to model interactions between gene sets (e.g. genome-wide gene–gene interactions [19], multidimensional variable set interactions [20], and multimodal dataset interactions [15]). Cui and Li [19] first proposed kernel interaction methods for genome-wide gene–gene interaction detection. Ge *et al.* [20] expanded on this concept to detect interactions between groups of genetic variants and sets of nongenetic factors, including disease risk factors, environmental exposures, and epigenetic markers. More recently, Alam [15] considered entire modal datasets as modeling units to identify interactions at the dataset level, and developed a higher-order interactions method (KMDHOI) to test interaction effects in multimodal datasets within the framework of a linear mixed effects model. Motivated by these advances, we developed a novel method named interactive multi-kernel learning (iMKL) grounded in a formal reproducing kernel Hilbert space (RKHS)-based functional ANOVA decomposition (see Durrande *et al.*, 2012 [21]). iMKL incorporates omics-omics interactions and heterogeneous data types within the framework of UMKL, to improve subtype identification. Our approach begins by constructing a sample-similarity kernel for each data type. Using these kernels, we propose a joint Hadamard product strategy to capture higher-order interactive effects between different omics types to generate interaction kernels. All the kernels are subsequently integrated into one meta-kernel under the UMKL framework. Based on this meta-kernel, *k*-means clustering is performed to identify cancer subtypes.

We applied iMKL to discover molecular subtypes in two types of renal cell carcinoma (RCC), clear cell renal cell carcinoma (ccRCC) and type II papillary renal cell carcinoma (type II pRCC), using datasets obtained from the TCGA website (http://cancergenome.nih.gov/). The results showed that iMKL outperformed the UMKL method with only marginal kernels. The molecular subtypes identified in ccRCC and type II pRCC patients, along with the subsequent biological analyses of key molecular features and pathways, provide novel insights into the prognostic value and biological significance for these cancers.

## Methods

### Interactive multi-kernel learning

**Figure 1:**
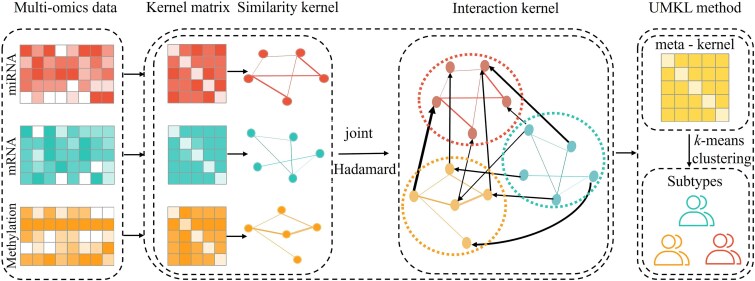
The flowchart of iMKL. The flowchart of iMKL begins by calculating the sample-similarity kernels for each omics data type, including miRNA, mRNA, and DNA methylation, termed marginal kernels. A joint Hadamard product strategy is then used to form interaction kernels from marginal kernels obtained with multi-omics data. All the marginal and interaction kernels are subsequently integrated into one meta-kernel under the UMKL framework, followed by the *k*-means clustering for subtype identification.

iMKL incorporates the interaction effect of multi-omics data types into a meta-kernel under the UMKL framework to improve subtype identification. It consists of three steps (See Fig. 1): (i) Constructing the sample-similarity kernel for each data type, termed the marginal kernel. We use a scaled exponential similarity kernel to construct a sample-by-sample similarity matrix for each data type. The matrix represents a similarity network in which the nodes correspond to samples, and the weighted edges represent the similarities between samples; (ii) Calculating the interaction kernels among different data types. Using the sample-similarity kernel of each data type from step (i), we model the paired interactions between different omics data types with a joint Hadamard product strategy; (iii) Integrating all the sample-similarity kernels from step (i) and (ii) to form a meta-kernel under the UMKL framework; and (iv) Cancer subtyping. *k*-means clustering is applied to cluster samples based on the meta-kernel for cancer subtyping.

Mathematically, our approach is grounded in a formal reproducing kernel Hilbert space (RKHS)-based functional ANOVA decomposition. Specifically, we employ the Hadamard product of kernels which corresponds to a product of base kernels defined over different input domains, i.e. different omics data types. For input $X=\left({X}_1,{X}_2,{X}_3\right)$, we define a kernel as:


$$ K\left(X,{X}^{\prime}\right)={K}^1\left({X}_1,{X}_1^{\prime}\right)\cdot{K}^2\left({X}_2,{X}_2^{\prime}\right)\cdot{K}^3\left({X}_3,{X}_3^{\prime}\right) $$

where each ${K}^i$ is a positive semi-definite kernel over the $i$th omics modality. According to the theory of ANOVA kernels (see Durrande *et al.*, 2012 [21]), the product kernel induces an RKHS that contains functions not only of the marginal effects ${f}_1 ({X}_1),{f}_2 ({X}_2),{f}_3 ({X}_3)$, but also their interactions, i.e.: 


\begin{eqnarray*} && f(X)={f}_0+{\sum}_l^3{f}_l\left({X}_l\right)+{f}_{12}\left({X}_1,{X}_2\right)+{f}_{13}\left({X}_1,{X}_3\right)+{f}_{23}\left({X}_2,{X}_3\right)\\&& \qquad +\ {f}_{123}\left({X}_1,{X}_2,{X}_3\right) \end{eqnarray*}

where $f$ is an unknown function on the product domain $\mathcal{X}={\mathcal{X}}_1\bigotimes{\mathcal{X}}_2\bigotimes{\mathcal{X}}_3$, and each component lies in a subspace of the full RKHS $\mathcal{H}$ which can be decomposed as:


$$ \mathcal{H}={\mathcal{H}}_1\oplus{\mathcal{H}}_2\oplus{\mathcal{H}}_3\oplus{\mathcal{H}}_{1\times 2}\oplus{\mathcal{H}}_{1\times 3}\oplus{\mathcal{H}}_{2\times 3}\oplus{\mathcal{H}}_{1\times 2\times 3} $$

where ${\mathcal{H}}_i$, ${\mathcal{H}}_{i\times j}$, ${\mathcal{H}}_{1\times 2\times 3}$ are RKHSs defined on ${\mathcal{X}}_i$, ${\mathcal{X}}_i\times{\mathcal{X}}_j$，and ${\mathcal{X}}_1\times{\mathcal{X}}_2\times{\mathcal{X}}_3$, respectively. The operator $\oplus$ denotes the direct sum, indicating orthogonal decomposition.

In iMKL, we first calculate the sample-similarity kernel for each omics data, such as miRNA, mRNA, and DNA methylation, termed marginal kernel. Suppose we have *M* types of omics data for *n* patients. The sample-similarity kernel is represented as a graph $G=\left(V,E\right)$. The vertices *V* correspond to the patients $\left\{{x}_1,{x}_2,\dots, {x}_n\right\}$ and the edges *E* are weighted by how similar the patients are. Edge weights are represented by an $n\times n$ sample-similarity kernel matrix $K$ with $K\left(i,j\right)$ indicating the similarity between patients ${x}_i$ and ${x}_j$. $K\left(i,j\right)$ is computed using a scaled exponential similarity kernel [11] as follows:


$$ K\left(i,j\right)=\mathit{\exp}\left(-\frac{\rho^2\left({x}_i,{x}_j\right)}{\mu{\varepsilon}_{i,j}}\right) $$

where $\rho \left({x}_i,\kern0.5em {x}_j\right)$ is the Euclidean distance between patients ${x}_i$ and ${x}_j$, *μ* is a hyperparameter that can be empirically set, ${\varepsilon}_{i,j}=\frac{mean\left(\rho \left({x}_i,{N}_i\right)\right)+ mean\left(\rho \left({x}_j,{N}_j\right)\right)+\rho \left({x}_i,{x}_j\right)}{3}$ which is utilized to address scaling issues, and $mean \left(\rho \left({x}_i,{N}_i\right)\right)$ is the average value of the distances between ${x}_i$ and each of its neighbors. The similarity kernel matrices for miRNA, mRNA, and DNA methylation are denoted as ${K}^1$, ${K}^2$, and ${K}^3$, respectively.

Using the sample-similarity kernel ${K}^1$, ${K}^2$, and ${K}^3$ for each data type, the paired interactive sample-similarity kernels are defined using a joint Hadamard product strategy:


$${K}^{1\times 2}={h}_{1\times 2}\left({X}_1,{X}_2\right)={K}^1\odot{K}^2$$

$${K}^{1\times 3}={h}_{1\times 3}\left({X}_1,{X}_3\right)={K}^1\odot{K}^3$$

$${K}^{2\times 3}={h}_{2\times 3}\left({X}_2,{X}_3\right)={K}^2\odot{K}^3$$

$${K}^{1\times 2\times 3}={h}_{1\times 2\times 3}\left({X}_1,{X}_2,{X}_3\right)={K}^1\odot{K}^2\odot{K}^3$$

where $\odot$ denotes the Hadamard product, representing the element-wise product of two matrices. ${K}^{1\times 2}$, ${K}^{1\times 3}$, ${K}^{2\times 3}$ represent pairwise omics-omics interactive kernels between different datasets, ${K}^{1\times 2\times 3}$ represents interactive kernel of three omics datasets.

We integrate all the sample-similarity kernel ${K}^1$, ${K}^2$, ${K}^3$, and the interaction kernels ${K}^{1\times 2}$, ${K}^{1\times 3}$, ${K}^{2\times 3}$, ${K}^{1\times 2\times 3}$ into one meta-kernel under the UMKL framework. UMKL constructs a *k*-nearest neighbor graph $G$, which is associated with each kernel. Then, an ($n\times n$)-matrix $K$, representing the topology of the data is defined such that ${K}_{ij}$ represents the number of times the pair $\left(i,j\right)$ is in the edge list of $G.$ Specifically, we introduce the *n*-dimensional vector ${\Delta }_i\left(\beta \right)$, ${\Delta }_i\left(\beta \right)=\left(\begin{array}{c}{K}_{i1}^{\ast}\\{}\vdots \\{}{K}_{in}^{\ast}\end{array}\right)$. We directly use the information provided by the different kernels through $K$ to measure the sample graph topology preservation. The following optimization problem is then solved:


$$ \underset{\beta }{\mathit{\min}}{\sum}_{i,j=1}^n{K}_{ij}{\left\Vert{\Delta }_i\left(\beta \right)-{\Delta }_j\left(\beta \right)\right\Vert}^2 $$

$$ \mathrm{for}\ {K}_{\beta}^{\ast }={\sum}_{l=1}^L{\beta}_l{K}^l $$

(1) \begin{equation*} \beta \in{\mathbb{R}}^L\ \mathrm{such}\ \mathrm{that}\ {\beta}_l\ge 0\ \mathrm{and}\ {\sum}_{l=1}^L{\beta}_l=1 \end{equation*}

where ${K}^l\left(l=1,\cdots, L\right)$ is the *l*th similarity matrix, *L* is the total number of similarity matrices, including the marginal and interaction kernel matrices.

For $M=3$ types of omics data, $L=7$, and ${\beta}_l$ is the weight of the *l*th similarity matrix. Equation (1) can be rewritten as:


$$ \underset{\beta }{\mathit{\min}}{\sum}_{l,{l}^{\prime }=1}^L{\beta}_l{\beta}_{l\prime }{K}^{l{l}^{\prime }} $$

(2) \begin{equation*} \mathrm{for}\ \beta \in{\mathbb{R}}^L\ \mathrm{such}\ \mathrm{that}\ {\beta}_l\ge 0\ \mathrm{and}\ {\sum}_{l=1}^L{\beta}_l=1 \end{equation*}

$$ \mathrm{for}\ {K}^{ll\prime }={\sum}_{i,j=1}^n{K}_{ij}\left\langle{\Delta }_i^l-{\Delta }_j^l,{\Delta }_i^{l^{\prime }}-{\Delta }_j^{l^{\prime }}\right\rangle $$

$$ \mathrm{and}\ {\Delta }_i^l=\left(\begin{array}{c}{K}_{i1}^l\\{}\vdots \\{}{K}_{in}^l\end{array}\right) $$

where ${K}^{l{l}^{\prime }}$ can be obtained by solving a standard Quadratic Programming (QP) problem in equation (2) and ${\beta}_l$ can be obtained through an ${L}_1$ constrained QP. This induces sparsity in the resulting kernel weights ${\beta}_l$, thereby effectively reducing redundancy among the kernels and mitigating the risk of overfitting (see also Speicher and Pfeifer, 2015 [22]). The final fused meta-kernel matrix is expressed as:


$$ {K}_{final}={\sum}_{l=1}^L{\beta}_l{K}^l,\mathrm{where}\ {\beta}_l\ge 0\ \mathrm{and}\ {\sum}_{l=1}^L{\beta}_l=1 $$

where ${\beta}_l$ is the weight of ${K}^l$, indicating the relative contribution of the corresponding kernel.

Based on the final integrated meta-kernel matrix ${K}_{final}$, we utilize *k*-means clustering [23] to obtain sample clusters. Assume that *k*-means divides all samples into *k* clusters: ${C}_1,{C}_2,\dots, {C}_k$. *k*-means can be described as minimizing the following objective function:


$$ E=\sum_{C=1}^k\sum_{x\in{C}_k}{\left\Vert{K}_{final}-{\mu}_k\right\Vert}_2^2 $$

where ${\mu}_k$ is the mean for ${C}_k$.

In our unsupervised learning framework, we construct the Hadamard product of individual kernels based on each omics data type. While the operation appears to act at the sample–similarity level, it is functionally equivalent to estimating functions in an RKHS that contains higher-order interaction components across omics sources. This connection to the ANOVA-RKHS formulation distinguishes our approach from additive kernel methods (which model only marginal effects).

### Estimating the optimal number of clusters

Estimating the number of clusters is a key step in disease subtyping. Eigengap [24] is used to determine the optimal number of clusters and is defined by identifying a significant drop in the magnitude of the eigenvalue gaps:


$$ eigengap(k)={\lambda}_{k+1}-{\lambda}_k $$

where ${\lambda}_k$ represents the $kth$ eigenvalue of the similarity Laplacian matrix, with the eigenvalues arranged in ascending order (${\lambda}_1\le{\lambda}_2\le \dots{\lambda}_n$). The optimal number of clusters ${C}^{\ast }$ is determined by locating the significant drop in the eigenvalue gaps:


$$ {C}^{\ast }=\underset{k>1}{\mathit{\max}}\, eigengap\, (k) $$

### TCGA datasets and data processing

We focused on subtypes of RCC, clear ccRCC and type II pRCC, using datasets downloaded from the TCGA website (http://cancergenome.nih.gov/). Detailed datasets and data processing for ccRCC and type II pRCC are provided in the supplementary files (Supplementary Note S1). After preprocessing, we obtained 285 ccRCC samples with 388 miRNAs, 16 893 mRNAs, and 10 994 methylation genes, as well as 67 type II pRCC samples with 437 miRNAs, 16 534 mRNAs, and 10 988 methylation genes.

### Downstream statistical analysis after subtyping

*Differential analysis.* We performed differential expression analysis for each omics data type across the identified subtypes. The Kruskal-Wallis H test was applied to identify DEmiRNAs, DEmRNAs, and DMGs, using a threshold of FDR-adjusted *P* < 0.05. The hypergeometric distribution [25] test was further used to identify features enriched in each subtype, with a filtering criterion of ${P}_{adj}<0.05$. Next, we conducted univariate Cox regression analysis for each differentially expressed gene to identify those associated with prognosis as candidate genes (${P}_{adj}<0.05$). Additionally, miRWalk [26] was used to predict the target genes of the identified DEmiRNAs.

#### Protein–protein interaction network analysis and hub genes identification

We conducted a protein–protein interaction (PPI) network analysis to further explore the hub genes associated with disease and their interactions. First, we downloaded the latest human PPI data from the Search Tool for the Retrieval of Interacting Genes (STRING) database (https://string-db.org/) with a high interaction confidence score (interaction score ≥ 0.7). The PPI network was then imported into Cytoscape 3.9.1 to visualize the relationships between protein-coding genes. Next, we used the CytoNCA [27] plugin in Cytoscape to calculate the Betweenness Centrality (BC) scores of candidate genes. The top 10 most highly connected genes, based on BC scores, were identified as the hub genes.

#### Hub genes functional annotation analysis

We performed GO [28] enrichment analysis and KEGG [29] pathway analysis on the identified hub genes using the R package clusterprofiler [30]. An FDR-adjusted *P <* 0.05 was used to determine whether a pathway was significantly impacted. GO and KEGG analyses offer complementary insights into the functions and roles of genes and proteins within biological systems.

#### Immune cell infiltration analysis and evaluation of the prognostic value of hub genes

We evaluated the tumor microenvironment by analyzing immune cell infiltration using the TIMER2.0 [31] online platform, a comprehensive database for immune cell analysis across multiple cancer types. The MCP-counter algorithms [32] were used. Differential immune cell infiltration across subtypes was assessed using the Kruskal-Wallis H test, with an FDR-adjusted *P* < 0.05. Lastly, we conducted a survival prognostic analysis of key genes to examine the impact of their expression levels on patient prognosis.

## Results

### Overall subtyping performance of ccRCC and type II pRCC

Based on the eigenvalue gaps criterion, iMKL identified three distinct subtypes of ccRCC, which exhibited significant differences in survival (*P* = 7.55E-04), as well as three subtypes of type II pRCC (*P* = 0.042) (See Table 1). Baseline clinical data for the different subtypes are provided in the supplementary materials (Supplementary Note S2), detailed in Table S1 and Table S2. Meanwhile, we conducted a comparative analysis between iMKL and subtyping approaches based on individual omics data types. As shown in Table S3, iMKL achieved log-rank $P$-values that were generally lower or comparable across both datasets, indicating favorable performance in survival stratification relative to subtyping based on individual omics data types (Supplementary Note S3). In addition, we also evaluated the clustering quality using both the silhouette score [33] and the Davies Bouldin Index (DBI) [34]. The results indicated that iMKL achieved higher silhouette scores (0.125 for ccRCC and 0.182 for type II pRCC) and lower DBI values (5.52 and 4.06, respectively) than UMKL, indicating improved clustering performance (Supplementary Note S4, Table S4, Figure S1).

**Table 1:** Comparison of subtyping results of different integration methods.

Cancer	ccRCC	type II pRCC
iMKL	**3 (7.55E-04)** ^**a**^	**3 (0.042)**
UMKL	3 (6.14E-03)	3 (0.375)
CIMLR	5 (1.32E-03)	4 (0.054)
PINSPlus	3 (2.07E-03)	2 (0.286)
LRAcluster	2 (1.92E-03)	3 (0.066)
IPFMC	3 (0.631)	3 (0.462)
iCluster	3 (1.60E-03)	3 (0.130)
iClusterBayes	2 (0.106)	3 (0.727)
SNF	3 (2.18E-04)	3 (0.011)
MOFA	3 (1.17E-03)	2 (0.231)

^a^The number of optimal subtypes identified by each method is listed, with the corresponding log-rank test *P*-value shown in parentheses. For iMKL, UMKL, IPFMC, SNF, and iCluster, the number of subtypes was determined based on the eigenvalue gaps. CIMLR selected the optimal number using separation cost, MOFA selected the optimal number using BIC criterion, iClusterBayes selected the optimal number of subtypes based on the CPI criterion, while PINSPlus automatically determined the best number of clusters. LRAcluster selected the optimal number of subtypes by maximizing the sum of the CPI [40] and gap statistic [41].

To better evaluate the method’s generalizability and performance, we compared iMKL with kernel-based methods that exclusively use marginal kernels, namely UMKL [13], CIMLR [25], as well as four state-of-the-art clustering methods, namely LRAcluster [7], PINSPlus [9], iCluster [10], IPFMC [35], SNF [11], MOFA [36], and iClusterBayes [37], for subtype identification in ccRCC and type II pRCC datasets. iMKL demonstrated more pronounced differences between subtypes and stronger separation in overall survival, achieving the smallest log-rank *P*-value among the methods. We then focused our comparison with UMKL, using the C-index [38, 39] as the metric for assessing the predictive accuracy in different cancer types. The C-index, ranging from 0.5 to 1, indicates perfect prediction at a score of 1 and random guessing at a score of 0.5. A higher C-index reflects better model performance, more details on the C-index are provided in the supplementary files (Supplementary Note S5). The C-index values for UMKL were 0.55 for ccRCC and 0.64 for type II pRCC, as shown in Table S5. In contrast, iMKL achieved superior performance, with C-index values of 0.61 for ccRCC and 0.73 for type II pRCC, representing improvements of 9.09% and 14.06%, respectively, compared to UMKL (Supplementary Note S6). These results indicate that iMKL outperforms UMKL in both subtype identification and predictive accuracy.

With multiple kernels assigned to each data type, iMKL automatically adjusts the kernel weights based on their contributions, assigning higher weights to kernels with greater information content and lower weights to those with less. Notably, the relative contributions of different kernels vary between ccRCC and type II pRCC (Supplementary Note S7). Compared with UMKL, iMKL can effectively capture the interaction effects between different omics data types (See Fig. S2). For ccRCC, the interaction between miRNA and mRNA (${K}^{1\times 2}$) has a higher weight in the final meta-kernel, indicating the important contribution of such interaction on ccRCC subtyping, while for type II pRCC, the weight for the interaction kernel between miRNA and promoter methylation (${K}^{1\times 3}$) is the highest indicating the importance of such interaction on type II pRCC subtyping. This capability provides valuable insights into the relative influence of each data type on molecular subtyping.

### Subtyping stability analysis

**Figure 2:**
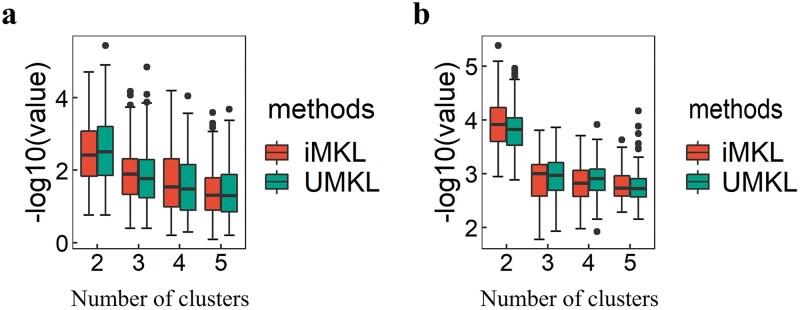
Boxplots of the -log10(*P*-values) obtained from the log-rank test comparing survival curves across different cluster numbers, using iMKL and UMKL, based on 100 random splits of ccRCC samples (a) and features (b).

**Figure 3:**
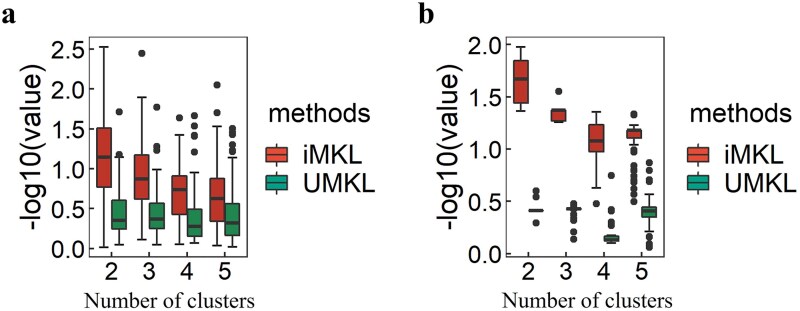
Boxplots of the -log10(*P*-values) obtained from the log-rank test comparing survival curves across different cluster numbers, using iMKL and UMKL, based on 100 random splits of type II pRCC samples (a) and features (b).

To further validate the robustness of the subtyping performance of iMKL and UMKL, we conducted stability analysis by randomly sampling patients or features in the ccRCC and type II pRCC datasets, following the methods described in [42]. Specifically, for ccRCC, we randomly sampled 50% of the patients and performed subtyping using iMKL and UMKL, assuming different numbers of clusters (e.g. 2–5). This process was repeated 100 times. For type II pRCC, 85% of the patients were sampled due to the smaller sample size. We also assessed robustness by sampling features. For ccRCC, we randomly sampled 80% of features in each omics dataset, while for type II pRCC, 85% were sampled, with the process repeated 100 times. For each sample or feature split, we applied a log-rank test to assess the survival differences between subtypes under the assumed number of clusters. The distribution of log-rank test *P*-values obtained using iMKL and UMKL are shown in Figs 2 and 3 for ccRCC and type II pRCC, respectively. Overall, iMKL demonstrated better performance compared to UMKL, though the difference was subtle for ccRCC. Under the condition of optimal subtyping number, the median *P-*values from 100 resampling runs for both ccRCC and type II pRCC were lower for iMKL compared to UMKL, indicating improved robustness and survival separation.

### Analysis of ccRCC subtypes identified by iMKL

**Figure 4:**
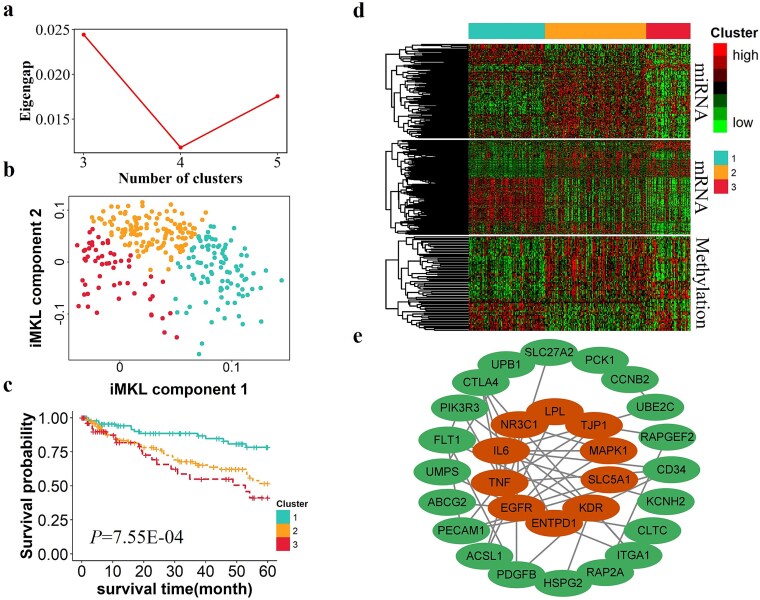
Clustering results of ccRCC. (a) Plot of eigengap (y-axis) showing 3 as the optimal number of clusters. (b) The 2-D visualization of the first two principal components (PCs) of three subtypes. (c) Kaplan–Meier survival curves of three subtype clusters obtained by iMKL. (d) The heatmaps of significant DEmiRNAs, DEmRNAs, and DMGs between different clusters. (e) PPI network containing 909 nodes and 732 edges.

We performed a subtype analysis of ccRCC patients to better understand the molecular heterogeneity and prognostic implications. The 285 patients were divided into three subtypes based on the eigenvalue gaps (See Fig. 4a). A 2-D visualization of these subtypes, derived from the final integrated meta-kernel matrix, effectively distinguished the three groups (See Fig. 4b). The survival curves, as shown in Fig. 4c, revealed significant differences between subtypes (log-rank *P* = 7.55E-04). To further explore the prognostic implications, we employed Cox regression models to estimate the association between different subtypes while adjusting for potential clinical covariates such as age, sex, and pathological stage. Before applying the model, we tested the proportional hazards (PHs) assumption using the Schoenfeld residuals method. The Global Schoenfeld Test yielded a *P-*value of 0.201, confirming that the PH assumption was satisfied, thereby ensuring the validity of the Cox model. Results from the Cox regression analysis (See Table 2) indicated that patients in cluster 3 had a 3.534-fold higher risk of death compared to cluster 1 (*P* = 4.62E-04), while patients in cluster 2 had a 2.103-fold higher risk of death compared to those in cluster 1 (*P* =0.022).

**Table 2:** Cox regression analysis of 285 ccRCC patients.

Variables	Coefficient (SE)	Wald	*P*-value	HR	95%CI
Subtypes					
Cluster 2^*^	0.743 (0.323)	2.299	**0.022**	2.103	(1.116, 3.963)
Cluster 3^*^	1.263 (0.36)	3.502	**4.62E-04**	3.534	(1.744, 7.165)
Gender	−0.225 (0.256)	−0.879	0.38	0.799	(0.483, 1.319)
Age	−0.235 (0.271)	−0.869	0.385	0.79	(0.465, 1.344)
Pathological stages					
Stage II	0.576 (0.542)	1.063	0.288	1.779	(0.615, 5.146)
Stage III^*^	1.603 (0.377)	4.257	**2.07E-05**	4.966	(2.374, 10.386)
Stage IV^*^	2.297 (0.352)	6.523	**6.91E-11**	9.946	(4.987, 19.834)

^a^Shows statistically significant (*P* < 0.05); Cluster 1 served as the reference for comparing distinctions among subtypes, while Stage I was employed as the reference for the comparison of differences across pathological stages; HR = Hazard Ratio.

### Differential expression and PPI network analysis for ccRCC

We identified a total of 1190 DEmRNAs, of which 400 were up-regulated and 790 were down-regulated; 146 DEmiRNAs, of which 78 were up-regulated and 68 were down-regulated; 72 abnormal DNA methylation genes, among which 37 were hypermethylated and 35 were hypomethylated. Figure 4d shows a heatmap of differential expression across different omics data, clearly illustrating distinct expression patterns among the three subtypes. Based on univariate Cox analysis of each differentially expressed gene, we identified several genes associated with prognosis as candidate genes, including 821 DEmRNAs and 53 DMGs. Furthermore, using the miRWalk online tool to predict the target genes of DEmiRNAs, we identified 66 DEmiRNAs that mapped to 47 target genes.

To further investigate the driver genes that may play a role in specific subtypes of ccRCC, a total of 921 candidate genes were used to construct a PPI network using STRING. The network contains 909 nodes and 732 edges using the criterion of an interaction confidence score ≥ 0.7 (Fig. 4e). The top hub genes identified by the BC analysis are likely to have significant roles in ccRCC subtype-specific pathways, with implications for therapeutic targeting. The top 10 node genes with the highest mediator were used as the hub genes for further analysis, namely *EGFR*, *TNF*, *IL6*, *NR3C1*, *LPL*, *TJP1*, *MAPK1*, *SLC5A1*, *KDR*, and *ENTPD1*. Figure 4e represents the interaction network of candidate genes and hub genes, with larger nodes and darker colors indicating higher BC values. We observed that the hub genes have the highest number of shortest paths passing through them, highlighting their critical role in facilitating inter-node communication. Notably, *KDR* plays a crucial role in angiogenesis and exhibits genetic interactions with multiple angiogenesis-related genes, contributing to tumor vascularization and drug resistance in ccRCC [43].

### Hub genes functional annotation analysis for clear renal cell carcinoma

**Figure 5:**
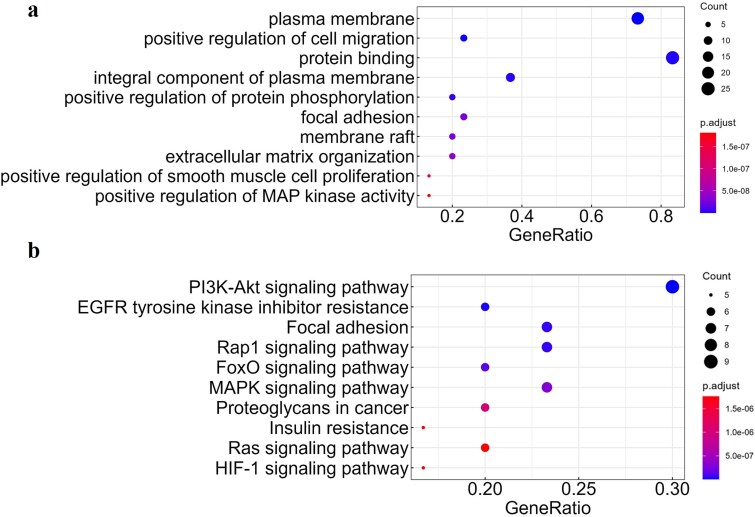
GO (a) and KEGG (b) enrichment analysis of key genes in ccRCC.

We then performed GO and KEGG pathway enrichment analysis on the top 30 genes with the highest BC values identified by the PPI network to explore the biological functions and pathways associated with key node genes. A total of 662 GO biological terms and 143 KEGG pathways were identified from the node genes of ccRCC. Figure 5 highlights the top 10 GO terms and KEGG pathways with the most significant differences. GO term analysis revealed that the genes were primarily involved in the composition of the cell membrane and its organic components and participated in essential biological processes such as cell migration, smooth muscle cell proliferation, protein phosphorylation, positive regulation of MAP kinase activity, and protein binding. RNA binding proteins (RBPs) serve critical functions in post-transcriptional regulation, significantly influencing the expression and function of oncoproteins and tumor suppressor proteins, which may partly explain the regulatory mechanisms underlying these biological processes [44]. KEGG pathway analysis indicated that the genes were mainly enriched in the PI3K-Akt signaling pathway, EGFR tyrosine kinase inhibitor resistance pathway, Rap1 signaling pathway, FoxO signaling pathway, MAPK signaling pathway, Ras signaling pathway, and HIF-1 signaling pathway. Notably, the excessive activation of PI3K-Akt and MAPK signaling pathways has been shown to be associated with the occurrence and progression of ccRCC [45]. Evidence suggests that HIF-1 is a key driver of ccRCC progression [46, 47], with a positive feedback loop between the PI3K-Akt and HIF-1 pathways contributing to ccRCC tumorigenesis [48]. The Rap1 signaling pathway plays a critical role in mediating cancer cell growth and apoptosis. Rap1 is highly expressed in ccRCC, positively correlates with pathological grade, and enhances the sensitivity of ccRCC to sunitinib treatment [49, 50].

### Immune cell infiltration analysis and evaluation of the prognostic value of hub genes

**Figure 6:**
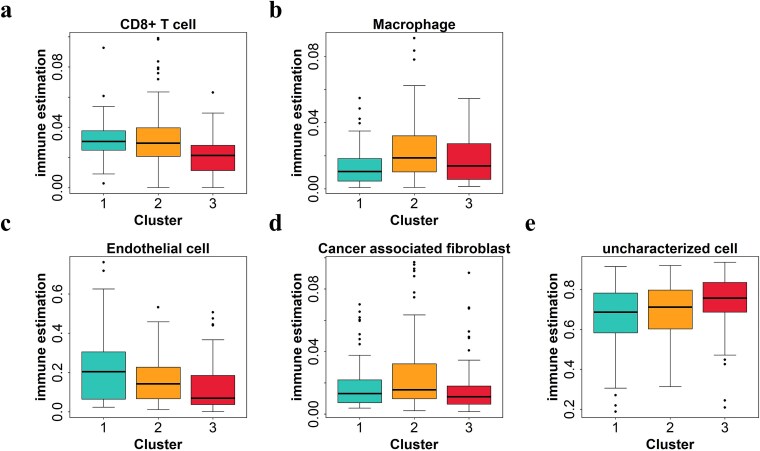
The difference of immune cell infiltration in different clusters of ccRCC. The abundance of CD8+ T cells, macrophage, endothelial cells, cancer-associated fibroblast, and uncharacterized cells in different clusters of ccRCC.

To better understand the biological implications of different molecular subtypes, we performed immune cell infiltration analysis for ccRCC. As shown in Fig. 6, five types of infiltrating cells including CD8+ T cells, macrophage, endothelial cells, cancer-associated fibroblast, and uncharacterized cells, exhibited significant differences between the three subtypes. Cluster 3 had lower levels of CD8+ T cells, cancer-associated fibroblast infiltration and endothelial cell infiltration, but higher levels of macrophages and uncharacterized cell infiltration compared to cluster 1, which was associated with a better prognosis. Studies have shown that the prognostic risk of tumor patients is negatively correlated with the level of endothelial cell infiltration [51]. The lack of CD8+ T cells in advanced and metastatic ccRCC may play a major role in resistance to available immune checkpoint therapies [52]. In both recurrent and locally invasive ccRCC, cancer-associated fibroblast infiltration should be considered a key cell type driving tumor progression and immunosuppression [53]. While the prognostic significance of macrophages has been confirmed in various tumors, their role as prognostic factors in ccRCC remains unclear. As key regulators of the tumor microenvironment, macrophages influence cancer progression, metastasis, and therapeutic response [54]. In contrast, high endothelial cell infiltration in KIRC was significantly associated with favorable prognosis, consistent with previous findings [55]. This observation also supports our current results.

**Figure 7:**
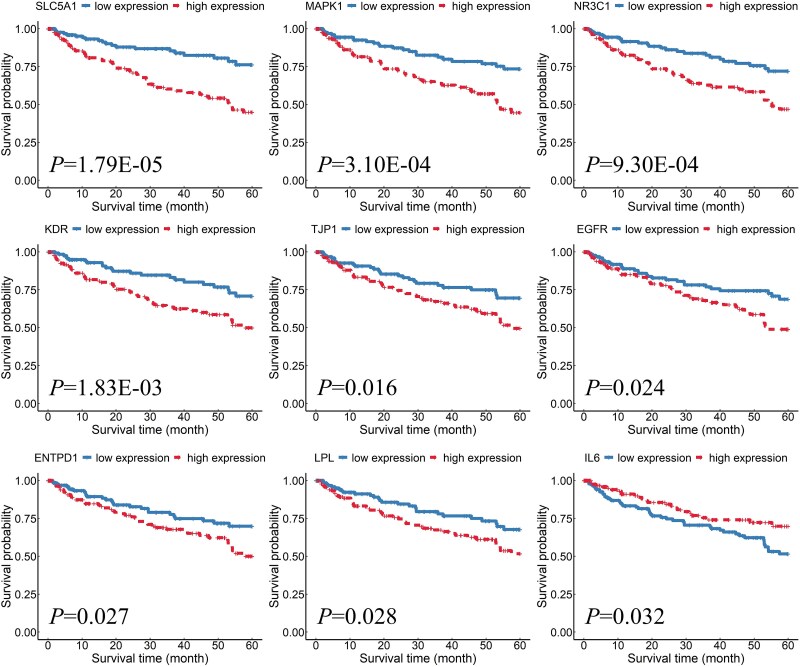
Plots showing prognostic survival curves of the nine hub genes for ccRCC sorted in ascending order by *P*-values.

To validate the prognostic value of the 10 hub genes, all patients were divided into two groups based on the median expression value of the hub genes: patients with expression levels above or equal to the median were classified as the high-expression group, while those below the median were classified as the low-expression group. The results showed that the expression levels of nine key genes (*SLC5A1*, *MAPK1*, *NR3C1*, *KDR*, *TJP1*, *EGFR*, *ENTPD1*, *LPL*, and *IL6*) were significantly correlated with patient prognosis. As shown in Fig. 7, except for *IL6*, the high-expression groups of *SLC5A1*, *MAPK1*, *NR3C1*, *KDR*, *TJP1*, *EGFR*, *ENTPD1*, and *LPL* were associated with poor prognosis. Bioinformatics analysis has identified *EGFR* as a novel key gene in ccRCC [56]. Increased expression of *NR3C1* has been observed in ccRCC biopsies from patients using immunohistochemistry [57, 58]. *MAPK1* has been found to be significantly active in advanced RCC, and its activity levels can predict the likelihood of metastasis in localized disease [59, 60]. *ENTPD1* has been reported to be associated with ccRCC. However, to the best of our knowledge, no research has yet linked *SLC5A1*, *LPL*, or *TJP1* to ccRCC [61].

### Analysis of type II pRCC subtypes identified by iMKL

We implemented similar analysis strategies for the type II pRCC data and identified three subtypes by iMKL. These subtypes were significantly associated with overall survival, with a log-rank *P*-value of 0.042 (Supplementary Fig. S3c). After adjusting for age, gender, and pathological grade, Cluster 1 patients had a 14.996-fold higher mortality risk than Cluster 2 patients (*P* =0.025) in the Cox regression analysis Supplementary Table S6 (Supplementary Note S8, Table S6). Due to space limitations, the details of the type II pRCC subtype analysis results are provided in the supplementary file (Supplementary Note S8).

### Robustness and adaptability of iMKL to other cancer subtyping

We also applied iMKL to the subtyping of low-grade gliomas (LGGs) and Breast Invasive Carcinoma (BRCA) using datasets obtained from the TCGA website. The results show that 499 LGG patients and 676 BRCA patients were each divided into two subtypes with significantly different prognoses ($P$-value =1.56E-14 for LGGs, $P$-value =1.68E-03 for BRCA), further demonstrating the robustness and adaptability of iMKL to other cancer subtyping tasks (Supplementary Note S9).

## Discussion

Cancer is a highly heterogeneous disease driven by complex molecular regulatory mechanisms. Numerous studies have shown that multi-omics data do not operate independently but interact across multiple levels in a highly interconnected manner. Taking these complex interactions into account is crucial for uncovering the molecular regulation mechanisms of cancer and further enhancing the identification of cancer subclusters. While recent multi-omics integration methods have predominantly aimed at capturing complementary information across various data types, few approaches specifically address intricate interactions between omics data types. To fill this gap, we proposed a novel method, iMKL, which incorporates omics-omics interactions alongside heterogeneous data types to enhance disease subtyping. Our evaluations highlight several key strengths of iMKL, including its superior clustering accuracy compared to state-of-the-art methods and its robustness in subtyping ccRCC and type II pRCC.

Our approach is innovative in several aspects. First, it captures higher-order interactions among different omics data types by leveraging a joint Hadamard product strategy to construct interactive sample-similarity kernels, based on the individual sample-similarity kernel of each data type. Secondly, iMKL uniquely integrates these interaction kernels together with the individual sample-similarity kernels, representing both omics-omics interactions and complementary information from diverse data types, into a single unified meta-kernel within an unsupervised learning framework. This approach automatically assigns weights to the kernels based on their informational contributions, enhancing the influence of high-information-content kernels while attenuating the impact of those with lower information content. In an application to two independent RCC datasets (ccRCC and type II pRCC), iMKL demonstrated its capability to identify biologically meaningful subtypes through comprehensive downstream statistical and bioinformatics analyses, outperforming other integration methods. Notably, iMKL exhibited superior stability in patient subtype identification compared to UMKL and effectively captured higher-order interactions with variable contributions of different omics data types. The identified subtypes revealed distinct differences in gene enrichment and immune cell infiltration, which are likely to play pivotal roles in the pathogenesis of ccRCC and type II pRCC. These findings further underscore the advantage of iMKL in integrative subtyping, offering valuable biological insights that could guide personalized treatment strategies.

In this study, we identified three distinct subtypes of ccRCC, each with unique prognostic implications. Specifically, patients in cluster 3 exhibited a 3.53-fold higher risk of mortality compared to those in cluster 1, while patients in cluster 2 faced a 2.1-fold increased risk. In addition, we provided a comprehensive biological interpretation of these subtypes, emphasizing their potential clinical relevance. Notably, we found significant differences in immune cell infiltration across the subtypes. KEGG pathway analysis indicated that activation of the PI3K-Akt pathway contributes to elevated phosphorylation levels, promoting tumor growth and progression in ccRCC [62]. Additionally, the FoxO signaling [63] and Rap1 signaling pathways [64] were reported to be closely associated with cancer tissue invasion and metastasis.

For type II pRCC, iMKL identified three distinct subtypes, with patients in cluster 1 experiencing a 15-fold higher risk of mortality compared to those in cluster 2. Similar to ccRCC, significant variations in immune cell infiltration were observed among the three subtypes, particularly involving B cells, cancer-associated fibroblasts, mononuclear macrophages, and endothelial cells. GO term analysis revealed that genes associated with these subtypes were primarily involved in biological processes such as cell division, protein binding, microtubule binding, and cell division spindle assembly. KEGG pathway analysis highlighted the pivotal role of genes in the p53 pathway in regulating apoptosis, genomic stability, and anti-angiogenesis, thereby mediating cell cycle arrest and apoptosis in type II pRCC [65, 66]. Moreover, we identified several hub genes associated with type II pRCC progression, including *CENPF*, which has been implicated as a prognostic biomarker in RCC, breast cancer, and bladder cancer [67–69].

Despite these findings, several limitations of our study must be acknowledged. First, our focus was limited to two highly heterogeneous RCC types: ccRCC and type II pRCC. Future work will extend the proposed iMKL method to additional cancer data to further validate its utility and expand its applicability. Moreover, incorporating pathway-based or network-based prior knowledge into iMKL holds promise for enhancing both biological interpretability and clustering performance. Additionally, while our study identified cancer-associated biomarkers through bioinformatics analyses, the causal relationship underlying these biomarkers remains unclear, necessitating further biological validation. Finally, regarding the evaluation of clustering performance, the widely used log-rank $p$-value assumes that if patient clusters exhibit significantly different survival outcomes, they are biologically distinct. Previous studies have reported that the log-rank $p$-value may yield inaccurate results when sample sizes are small or cluster sizes are unbalanced [4]. Therefore, additional metrics such as the silhouette score and Davies–Bouldin Index should be considered to complement the assessment of clustering performance.

In conclusion, the proposed iMKL method effectively captures potential omics-omics interactions for multi-omics data integration, achieving robust and superior accuracy in subtype identification. It provides a novel computational tool for cancer subtype discovery, as demonstrated by the identification of ccRCC and type II pRCC subtypes, which exhibit distinct prognostic molecular characteristics. These findings offer additional biological and clinical insights into ccRCC and type II pRCC. Furthermore, iMKL holds significant potential for application to other heterogeneous diseases, which are prevalent in biomedical research.

**Key Points**

- We proposed a novel method, iMKL, which leverages omics-omics interactions alongside different omics data types to improve disease subtyping.
- iMKL captures higher-order interactions between various omics data types by Hadamard products of sample-similarity kernels of individual omics data and further forms a meta-kernel under the unsupervised kernel learning framework for subsequent disease subtyping.
- iMKL demonstrated superior performance in patient subtyping compared to UMKL.
- When applied to two RCC datasets, iMKL identified biologically meaningful subtypes followed by comprehensive downstream statistical and bioinformatics analyses, revealing superior performance over other integration methods.

## Supplementary Material

Supplementary_materials_bbaf687

## Data Availability

The ccRCC and pRCC datasets were obtained from the TCGA website (http://cancergenome.nih.gov/).
