# Supplementary material for: Multi-omics data integration for enhanced cancer subtyping via interactive multi-kernel learning
Source: Brief Bioinform. 2025 Dec 17;26(6):bbaf687. doi: 10.1093/bib/bbaf687 (PMC12710476; doi:10.1093/bib/bbaf687)
Supplement: Supplementary_materials_bbaf687 [file supplementary_materials_bbaf687.docx]

**^[[1]](#footnote-1)^**  Supplemental materials for **“Multi-omics data integration for enhanced cancer subtyping via interactive multi-kernel learning”**

Hongyan Cao^a,b,*^, Tong Wang^a,c^, Zhaoyang Xu^a,c^, Xin Zhao^a^, Gaiqin Liu^a,b^, Xiaoling Yang^d^, Ruiling Fang^a,b^, Yanhong Luo^a,b^, Ping Zeng^e^, Hongmei Yu^a,b^, Yanbo Zhang^a,b^, Yuehua Cui^f,*^

^a^*Department of Health Statistics*, *Shanxi Provincial Key Laboratory of Major*

*Diseases Risk Assessment*, *School of Public Health*, *Shanxi Medical University*,

*Taiyuan*, *Shanxi 030001*, *PR China*

^b^*MOE Key Laboratory of Coal Environmental Pathogenicity and Prevention*,

*Shanxi Medical University*, *Taiyuan*, *Shanxi 030001*, *PR China.*

^c^*Academy of Medical Sciences*, *Shanxi Medical University*, *Taiyuan, Shanxi*

*030001*, *PR China*

^d^*Department of Thoracic Oncology*, *Shanxi Bethune Hospital, Shanxi Academy*

*of Medical Sciences*, *Tongji Shanxi Hospital*, *Third Hospital of Shanxi Medical*

*University*, *Taiyuan*, *Shanxi 030032*, *PR China*

^e^*Department of Biostatistics, School of Public Health, Xuzhou Medical University,*

*Xuzhou, Jiangsu 221004, PR China*

^f^*Department of Statistics and Probability, Michigan State University, East Lansing,*

*MI 48824, USA*

**1. Supplementary Note 1: TCGA datasets and data processing**

We focused on subtypes of renal cell carcinoma (RCC). According to the latest assessment by the International Agency for Research on Cancer (IARC) of the World Health Organization in 2022, kidney tumors are among the most prevalent forms of malignancies in the urinary system, with increasing incidence and mortality worldwide [1]. RCC refers to cancer originating from the renal epithelium, accounting for approximately 90% of kidney cancers [2]. Traditionally, RCC is classified histopathologically into three main categories: clear cell renal cell carcinoma (ccRCC, 75%), papillary renal cell carcinoma (pRCC, 15-20%), and chromophobe renal cell carcinoma (chRCC, 5%). pRCC is further divided into type Ⅰ and type Ⅱ based on histological criteria. Compared with type Ⅰ pRCC, type Ⅱ pRCC exhibits greater molecular heterogeneity and worse clinical prognosis [3-4]. Consequently, our study primarily focuses on subtyping ccRCC and type Ⅱ pRCC.

The ccRCC and pRCC datasets were downloaded from the TCGA website using the TCGAbiolinks R package [5]. Three types of omics data were considered, miRNA expression, mRNA expression, and promoter CpG methylation. For type Ⅱ pRCC, we selected samples classified as tumor type II to serve as the type II pRCC dataset. The ccRCC and type Ⅱ pRCC datasets were preprocessed as follows:

1) Promoter CpG methylation annotation: Annotated CpG methylation sites within 2 kbp of the transcription start site [6]. CpG sites located on sex chromosomes were removed. The remaining CpG sites were mapped to genes, and the mean beta value of multiple CpG sites per gene was used as the gene-level methylation signal.

2) Feature filtering and imputation: Features with a deletion ratio greater than 30% were removed. Missing values in the remaining data were imputed using the K-nearest neighbor (KNN) algorithm [7].

3) Data transformation: miRNA and mRNA data were transformed using a ${log}_{2}(x+1)$ conversion.

After preprocessing, we obtained 285 ccRCC samples with 388 miRNAs, 16,893 mRNAs, and 10,994 methylation genes, as well as 67 type II pRCC samples with 437 miRNAs, 16,534 mRNAs, and 10,988 methylation genes.

**2. Supplementary Note 2: Baseline clinical data for different subtypes of ccRCC and Type II pRCC Patients**

We performed subtype identification on the ccRCC and type Ⅱ pRCC datasets. Baseline clinical data for the identified subtypes are shown in Tables S1 and S2, detailing demographic and clinical characteristics such as age, gender, pathology, and survival status.

**Table S1** Baseline clinical data for different subtypes of ccRCC patients.

| Item | Cluster 1 | Cluster 2 | Cluster 3 |
| --- | --- | --- | --- |
| Number of patients (*n*, %) | 98(34.39) | 130(45.61) | 57(20.00) |
| Age (year, mean ± SD) | 59.39±11.00 | 60.40±10.30 | 60.03±10.61 |
| Gender (*n*, %) |  |  |  |
| Male | 61(62.24) | 93(71.54) | 31(54.39) |
| Female | 37(37.76) | 37(28.46) | 26(45.61) |
| Pathological stages (*n*, %) |  |  |  |
| Stage Ⅰ | 54(55.10) | 51(39.23) | 33(57.89) |
| Stage Ⅱ | 14(14.29) | 10(7.69) | 5(8.77) |
| Stage Ⅲ | 21(21.43) | 36(27.69) | 7(12.28) |
| Stage Ⅳ | 9(9.18) | 33(25.38) | 12(21.05) |
| Survival status (*n*, %) |  |  |  |
| Alive | 84(85.71) | 89(68.46) | 38(66.67) |
| Dead | 14(14.29) | 41(31.54) | 19(33.33) |

**Table S2** Baseline clinical data for different subtypes of type Ⅱ pRCC patients

| Item | Cluster 1 | Cluster 2 | Cluster 3 |
| --- | --- | --- | --- |
| Number of patients (*n*, %) | 31(42.26) | 19(28.36) | 17(25.38) |
| Age (year, mean ± SD) | 58.70±13.40 | 64.57±9.95 | 67.94±5.71 |
| Gender (*n*, %) |  |  |  |
| Male | 18(58.06) | 12(63.16) | 15(88.24) |
| Female | 13(41.94) | 7(36.84) | 2(11.76) |
| Pathological stages (*n*, %) |  |  |  |
| Stage Ⅰ | 15(48.40) | 9(47.37) | 13(76.47) |
| Stage Ⅱ | 1(3.22) | 3(15.80) | 1(5.88) |
| Stage Ⅲ | 14(45.16) | 6(31.57) | 3(17.65) |
| Stage Ⅳ | 1(3.22) | 1(5.26) | - |
| Survival status (*n*, %) |  |  |  |
| Alive | 24(77.42) | 18(94.74) | 16(94.12) |
| Dead | 7(22.58) | 1(5.26) | 1(5.88) |

**3. Supplementary Note 3: Comparison of subtyping results obtained from multi-omics and single-omics data**

We conducted a comparative analysis between iMKL and subtyping approaches based on individual omics data types. As shown in Table S3, iMKL achieved log-rank $P$-values that were generally lower or comparable across both datasets, indicating favorable performance in survival stratification relative to subtyping based on individual omics data types.

**Table S3.** Comparison of survival analysis between different subtypes obtained with multi-omics and single omics data

|  | multi-omics | miRNA | mRNA | methylation |
| --- | --- | --- | --- | --- |
| ccRCC | 3(7.55E-04) | 2(1.68E-04) | 2(7.06E-04) | 2(0.49) |
| type Ⅱ pRCC | 3(0.042) | 2(0.637) | 2(0.083) | 3(0.042) |

**4. Supplementary Note 4: Evaluation of clustering quality using silhouette scores and the** **Davies Bouldin index**

We evaluated the clustering quality using both the silhouette score [8] and the Davies-Bouldin Index (DBI) [9], The silhouette score reflects cluster cohesion and separation, whereas the DBI evaluates the balance between intra-cluster compactness and inter-cluster separation, where lower values indicate better clustering performance. As shown in Table S4, iMKL achieved higher silhouette scores (0.125 for ccRCC and 0.182 for type II pRCC) and lower DBI values (5.52 and 4.06, respectively) than UMKL, indicating improved clustering performance. The corresponding silhouette plots are provided in Figure S1.

**Table S4**. The clustering performance of iMKL and UMKL on ccRCC and type Ⅱ pRCC

|  |  | iMKL | UMKL |
| --- | --- | --- | --- |
| ccRCC | Silhouette | **0.125** | 0.123 |
|  | DBI | **5.52** | 5.73 |
| type Ⅱ pRCC | Silhouette | **0.182** | 0.152 |
|  | DBI^*^ | **4.06** | 4.67 |

Note: ^*^DBI denotes the Davies Bouldin Index; lower values indicate better clustering performance.


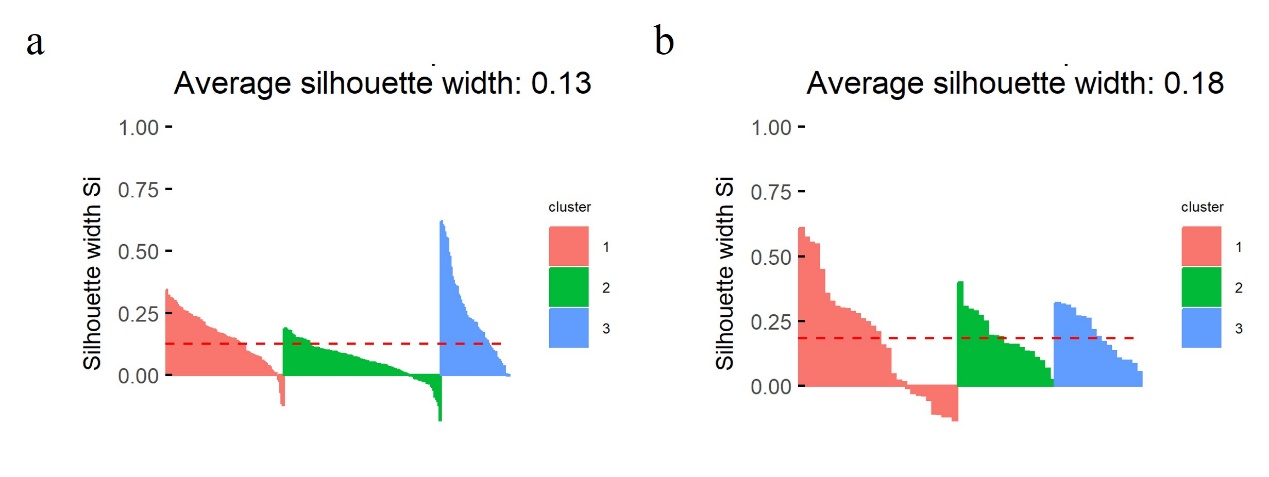


Figure S1. Silhouette width plots of the subtypes identified by iMKL for ccRCC (a) and type II pRCC (b).

Alt text: Silhouette width plots displaying the distribution of silhouette values for clusters identified by iMKL in ccRCC and type II pRCC.

**5. Supplementary Note 5: The C-index metric**

The C-index is defined as the ratio of the number of concordant predictions (survival times or survival probabilities) over the number of possible pairs of observed survival times [10-11]. The formula for computing the C-index is given as follows:

$$C=\frac{1}{n}\sum_{i=1}^{n} I(z_{i}>z_{j}\mid t_{i}<t_{j})$$

Here, $n$ denotes the number of all possible pairs $(i,j)$ such that $z_{i}\neq z_{j}$, meaning the individuals have different predicted risks and survival times. $z_{i}$ and $z_{j}$ represent the predicted risks, while $t_{i}$ and $t_{j}$ are their actual survival times. The indicator function $I$ equals 1 if the condition inside the parentheses is true (i.e., individual $i$ has a shorter survival time than individual $j$, and the predicted risk of$i$ is greater than that of $j$), and 0 otherwise.

Note that, C-index=1 indicates the model has a perfect prediction, and C-index=0.5 implies that the model is as good as a random predictor. Usually, a larger C-index implies a better performance of the model.

**6. Supplementary Note 6: Comparison of subtype analysis results in ccRCC and type II pRCC using UMKL and iMKL**

We focused our comparison on UMKL, using the C-index as the metric to evaluate predictive accuracy across various methods and cancer types. The C-index, which ranges from 0.5 to 1, signifies perfect prediction at a score of 1, while a score of 0.5 indicates random guessing. As shown in Table S5, the C-index values for UMKL were 0.55 for ccRCC and 0.64 for type II pRCC. In contrast, iMKL demonstrated superior performance, achieving C-index values of 0.61 for ccRCC and 0.73 for type II pRCC, reflecting improvements of 9.09% and 14.06%, respectively. These results indicate that the iMKL method outperforms UMKL in both subtype identification and predictive accuracy.

**Table S5** Subtype analyses on ccRCC and type Ⅱ pRCC.

| Item | | ccRCC | | | |  | | type Ⅱ pRCC | | |  |
| --- | --- | --- | --- | --- | --- | --- | --- | --- | --- | --- | --- |
|  |  | UMKL | | iMKL | |  | | UMKL | iMKL | |  |
| Number of clusters |  | | 3 | | 3 | |  | 3 | | 3 | |
| C-index |  | | 0.55 | | **0.61** | |  | 0.64 | | **0.73** | |

**7. Supplementary Note 7: Relative subtyping contributions of different omics data types with iMKL in ccRCC and Type II pRCC**

With multiple kernels assigned to each data type, the iMKL method automatically optimizes weights for different kernels based on their contributions, up-weighting the kernels with higher information content while down-weighting those with lower information content. Notably, the relative contributions of different kernels vary between ccRCC and type Ⅱ pRCC. Compared with UMKL, iMKL more effectively captures the higher-order interaction from different omics data types. This capability provides valuable insights into the relative influence of each data type and their interactions on molecular subtyping (See Figure S2). For example, for ccRCC, the interaction between miRNA and mRNA ($K^{1\times2})$ has a higher weight in the final meta-kernel, indicating the important contribution of such interaction on ccRCC subtyping, while for type II pRCC, the weight for the interaction kernel ($K^{1\times3})$ between miRNA and promoter methylation is the highest, indicating the importance of such interaction on type II pRCC subtyping.


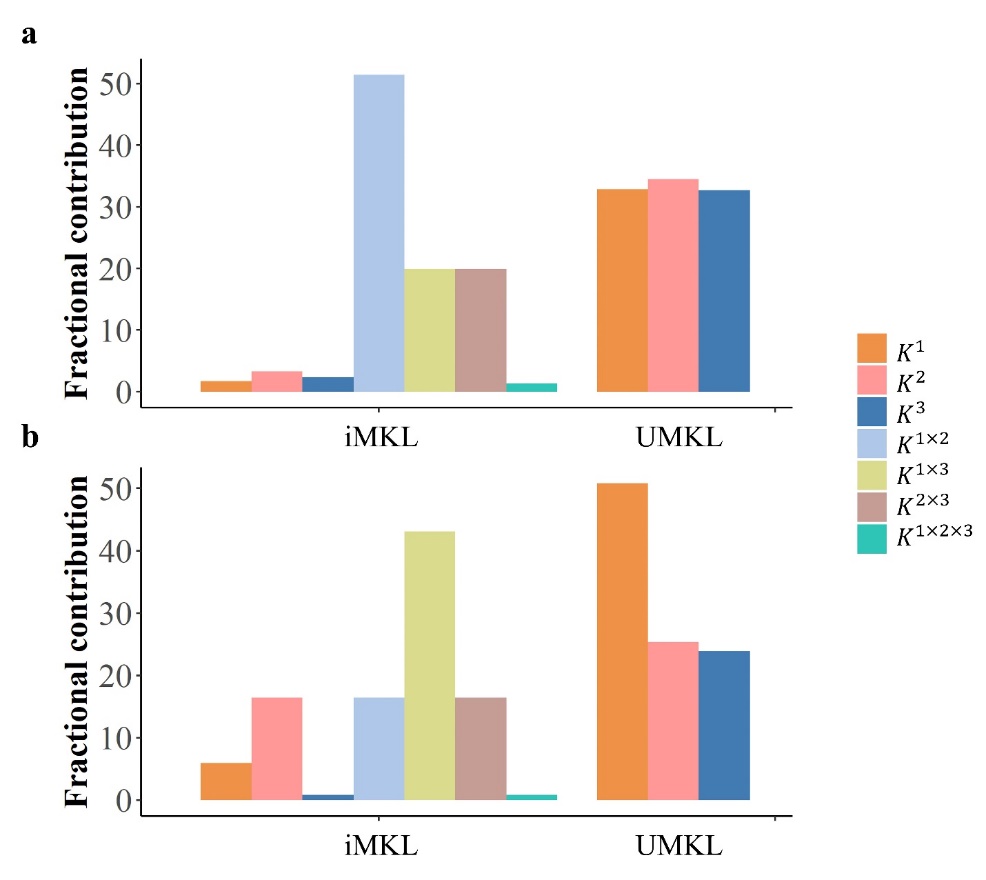


**Figure S2.** Relative contributions of different data types for subtyping with iMKL and UMKL in ccRCC and type Ⅱ pRCC. The height of each bar represents the fraction of kernel weight for each data type in ccRCC (a) and type Ⅱ pRCC (b). Specifically, the marginal kernels for miRNA, mRNA, and DNA methylation are denoted as $K^{1}$, $K^{2}$, and $K^{3}$, respectively.

Alt text: Barplots showing the relative kernel weight contributions of different omics data types for iMKL and UMKL in ccRCC and type II pRCC.

**8. Supplementary Note 8: Real data analysis on Type II pRCC**

**8.1 Analysis of** **type Ⅱ pRCC subtypes identified by iMKL**

We applied iMKL to type Ⅱ pRCC patients to achieve subtyping. The 67 Patients were divided into three subtypes based on the eigenvalue gaps (Figure S3a). A 2-D visualization of these subtypes, derived from the final integrated meta-kernel matrix, effectively distinguished the three groups (See Figure S3b). The survival curves, as shown in Figure S3c, revealed significant differences between the subtypes (log-rank *P*-value=0.042). Cox regression analysis was applied while controlling for the effects of covariates such as age, gender, and pathological grade. As shown in Table S6, the mortality risk of patients in cluster 1 was 14.996 times higher than that of patients in cluster 2 (*P-*value=0.025).


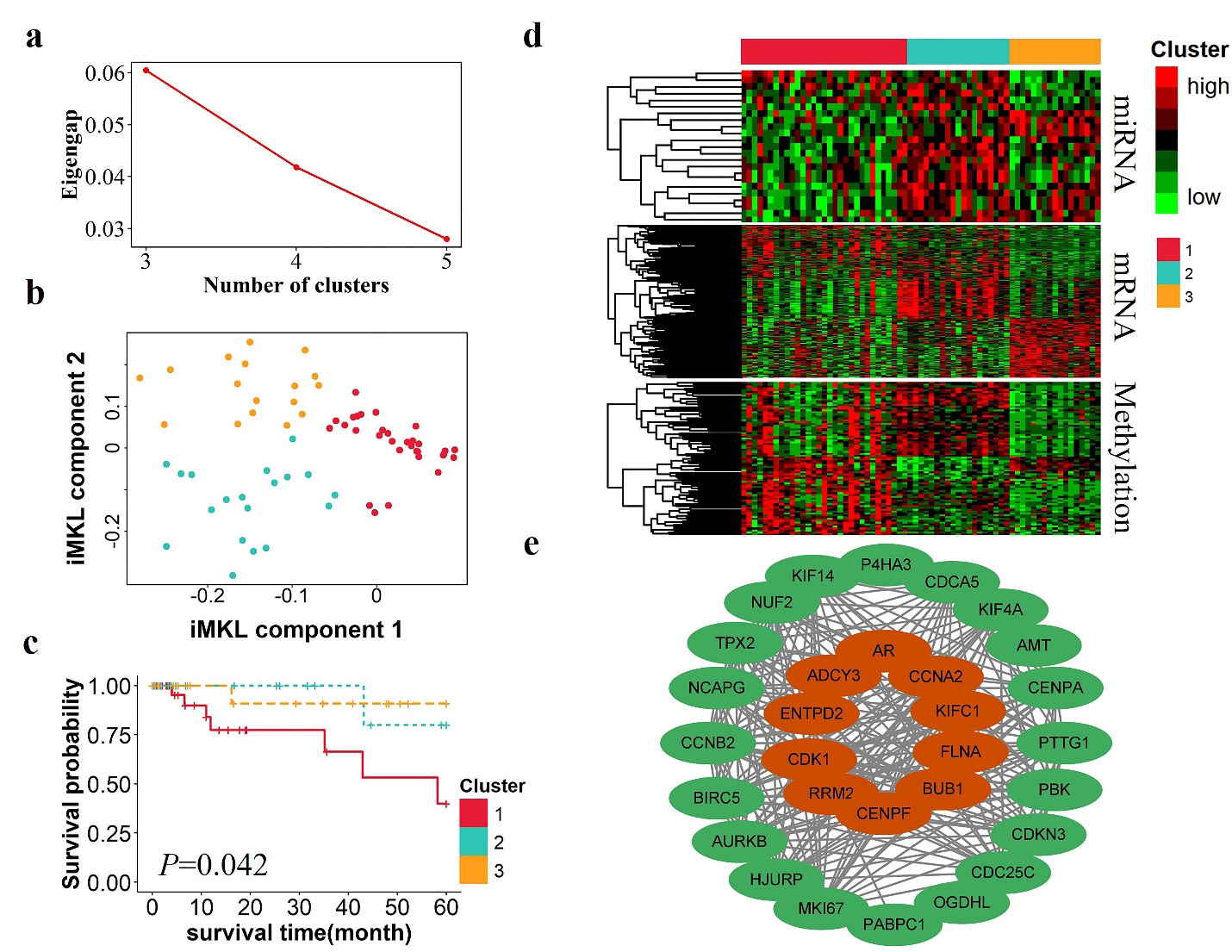


**Figure S3.** Clustering results of type Ⅱ pRCC. (a) Plot of eigengap (y-axis) showing 3 as the optimal number of clusters. (b) The 2-D visualization of the first two principal components (PCs) of three subtypes. (c) Kaplan-Meier survival curves of three clusters obtained by iMKL. (d) The heatmaps of DEmiRNAs, DEmRNAs, and DMGs between different clusters. (e) Protein-protein interaction (PPI) network containing 234 nodes and 267 edges.

Alt text: A multi-panel figure showing an eigengap line plot, a two-dimensional PCA scatter plot of clusters, Kaplan–Meier survival curves, heatmaps of multi-omics features, and a protein-protein interaction network for type Ⅱ pRCC.

**Table S6.** Cox regression analysis of 67 type Ⅱ pRCC patients

| Variables | Coefficient (SE) | Wald | *P*-value | HR | 95%CI |
| --- | --- | --- | --- | --- | --- |
| Subtypes |  |  |  |  |  |
| Cluster 1* | 2.708(1.212) | 2.234 | **0.025** | 14.996 | (1.394,161.273) |
| Cluster 3 | 1.617(1.672) | 0.967 | 0.334 | 5.039 | (0.190,133.594) |
| Gender | 0.465(0.872) | 0.533 | 0.593 | 1.592 | (0.288,8.794) |
| Age | -2.162(1.223) | -1.767 | 0.077 | 0.115 | (0.010,1.266) |
| Pathological stages |  |  |  |  |  |
| Stage Ⅱ | 2.477(1.698) | 1.458 | 0.145 | 11.899 | (0.427,331.835) |
| Stage Ⅲ* | 3.021(1.426) | 2.118 | **0.034** | 20.516 | (1.254,335.758) |
| Stage Ⅳ* | 5.280(1.770) | 2.983 | **0.003** | 196.37 | (6.114,6307.482) |

*Shows statistically significant (*P*<0.05); Cluster 2 served as the reference for comparing distinctions among subtypes, while Stage I was employed as the reference for the comparison of differences across pathological stages; HR=Hazard Ratio.

**8.2 Differential expression and PPI network analysis for type II pRCC**

Through differential expression analysis of the type II pRCC dataset, 867 differentially expressed mRNAs (DEmRNAs) were identified, comprising 412 up-regulated and 455 down-regulated genes. Additionally, 23 differentially expressed miRNAs (DEmiRNAs) were identified, including 6 up-regulated and 17 down-regulated miRNAs. For DNA methylation, 82 differentially methylated genes (DMGs) were identified, of which 35 were hypermethylated and 47 were hypomethylated. Figure S3d illustrates a heatmap of the differentially expressed genes across different omics, where rows represent features and columns represent samples. Red indicates relatively high expression, while green represents low expression. The heatmap highlights significant heterogeneity in the expression profiles of type II pRCC across the three patient subgroups, underscoring the complexity of the disease. To further investigate the genes influencing prognosis, univariate Cox regression analysis was performed on the differential genes, resulting in 235 characteristic genes associated with the prognosis of type II pRCC. These included 204 DEmRNAs and 31 DMGs.

To identify key genes potentially involved in type II pRCC subtypes, we constructed a protein-protein interaction (PPI) network using the 235 candidate genes via the STRING database. With an interaction score threshold of 0.7, the PPI network comprised 234 nodes and 267 edges. Betweenness centrality (BC) analysis revealed that *RRM2, CDK1, ENTPD2, ADCY3, AR, CCNA2, KIFC1, FLNA, BUB1*, and *CENP* exhibited the highest BC values. These genes appear to play pivotal roles in the molecular network and may act as key regulators of type II pRCC subtypes (See Figure S3e). Among these hub genes, abnormal expression of *BUB1* may disrupt chromosomal replication fidelity and induce aneuploidy, thereby facilitating tumor development[12]. *CDK1* functions as a key regulator of cell cycle progression and survival, and its elevated expression has been observed in various malignancies. Notably, increased CDK1 activity has been linked to RCC recurrence and unfavorable prognosis across multiple cancer types, including ovarian, colorectal, and breast cancers[13].

**8.3 Hub genes functional annotation analysis for type II pRCC**

To further understand the functional characteristics of key genes, GO biological function annotation and KEGG pathway enrichment analysis were performed on 30 genes within the PPI network. A total of 362 GO biological function terms and 66 KEGG pathways were enriched. Figure S4 highlights the top 10 GO terms and KEGG pathways with the most significant enrichment. GO analysis revealed that the key genes were mainly involved in biological processes such as cell division, protein binding, microtubule binding, and cell division spindle assembly. Additionally, they were associated with critical cellular components, including the cytosol, nucleus, cell membrane, nucleoplasm, midbody, and the centromere region of chromosomes. KEGG analysis showed significant enrichment of key genes in the p53 signaling pathway, human T-cell leukemia virus type 1 infection, and human immunodeficiency virus type 1 infection. Moreover, these genes were also implicated in processes such as oocyte maturation, meiosis, purine metabolism, and cellular senescence. The p53 gene is the most frequently mutated gene in human cancers and has been extensively studied across a wide range of malignancies. As a tumor suppressor, p53 plays a critical role in promoting apoptosis by activating the expression of the pro-apoptotic protein Bax and suppressing the anti-apoptotic protein Bcl-2 [14]. In addition, alterations in metabolic pathways-ranging from glycolysis to mitochondrial function-are also evident in RCC. These metabolic reprogramming events not only influence tumor cell functionality but also reshape the local tumor microenvironment, thereby creating potential vulnerabilities for therapeutic intervention[15].


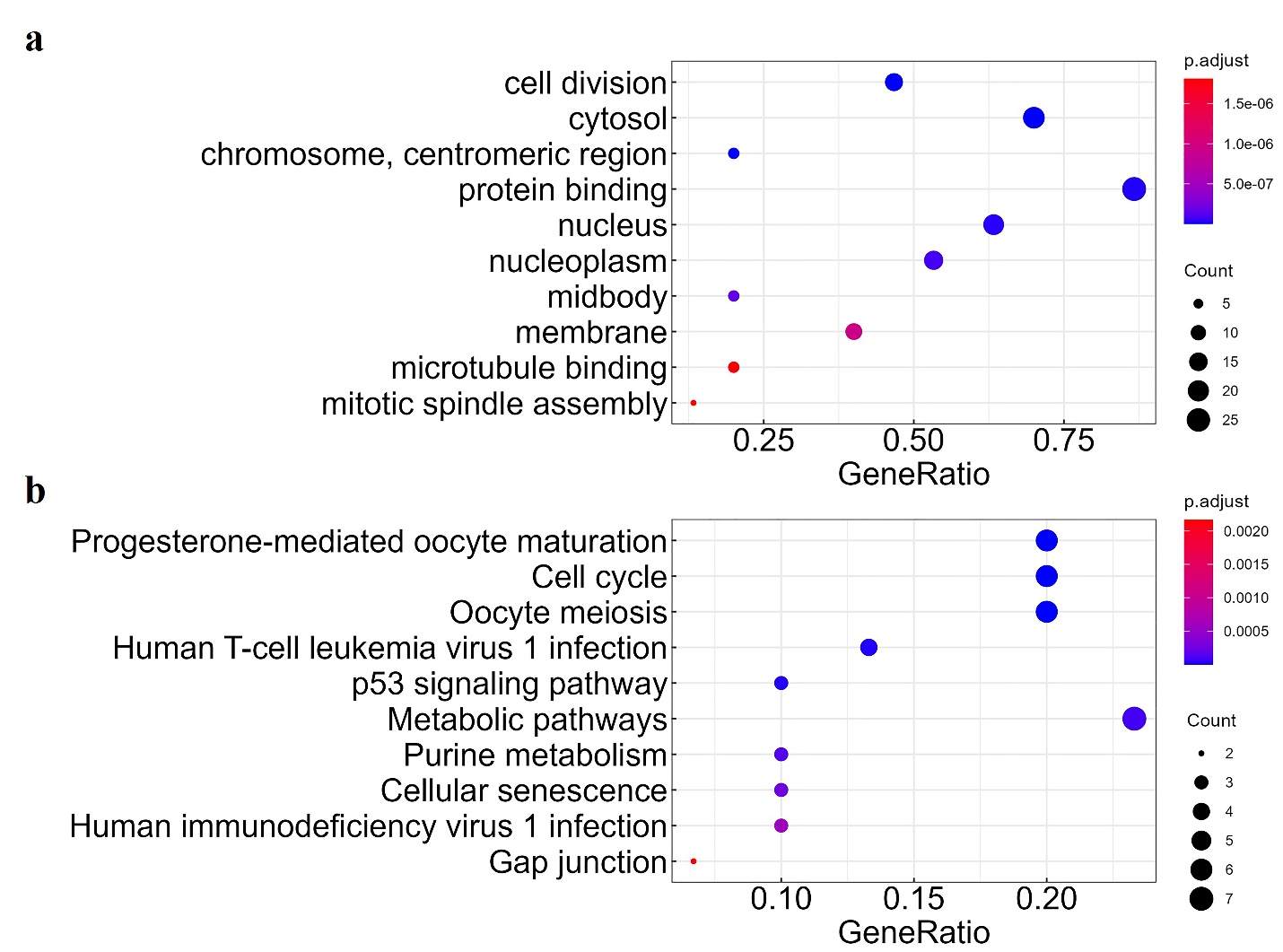


**Figure S4.** GO (a) and KEGG (b) enrichment analysis of key genes in type Ⅱ pRCC.

Alt text: Two enrichment plots displaying Gene Ontology and KEGG pathway results for key genes in type Ⅱ pRCC.

**8.4 Immune cell infiltration analysis and evaluation of the prognostic value of hub genes**

As shown in Figure S5, significant differences were observed in the infiltration levels of four immune cell types: B cells, cancer-associated fibroblasts, mononuclear macrophages, and endothelial cells, across the three subtypes of type Ⅱ pRCC. In Cluster 1, endothelial cells and cancer-associated fibroblasts exhibited the highest infiltration abundance, while mononuclear macrophages and B cells showed lower infiltration levels compared to Cluster 2, which had the best prognosis. Macrophages, the most abundant cells in the tumor microenvironment, are known to drive the progression of several malignant tumors, including breast cancer and lung cancer [16, 17]. The development of pRCC is closely linked to the VEGF axis signal in tumor and endothelial cells. Targeted vascular endothelial growth factor receptor has anti-tumor activity [18], and cancer-associated fibroblasts play a key role in promoting the development of renal cell carcinoma [19]. This study is the first to propose specific tumor microenvironment infiltrating cells, such as B cells and cancer-associated fibroblasts, as potentially associated with type II pRCC. However, their causal relationships with type Ⅱ pRCC progression require further biological validation.


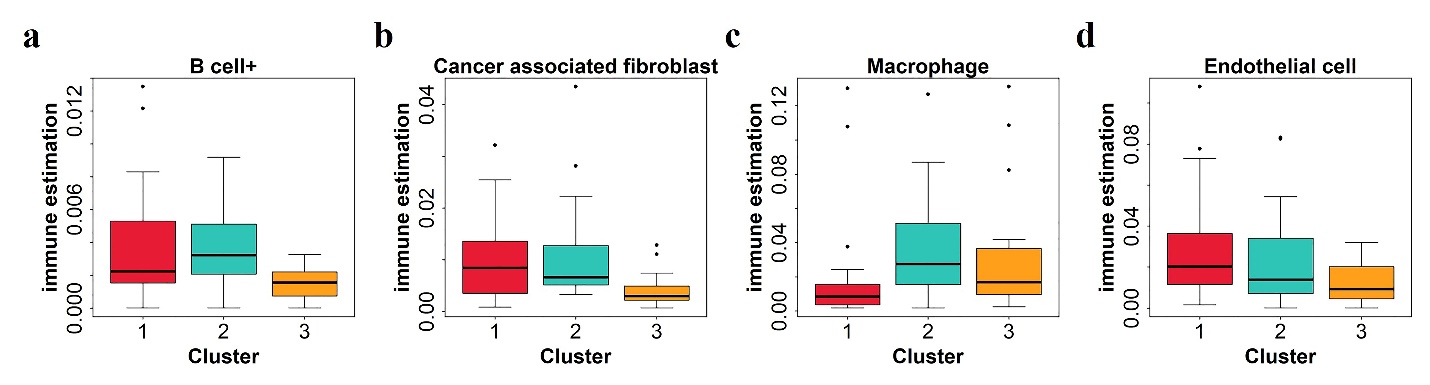


**Figure S5.** Immune cell infiltration differences across clusters of type Ⅱ pRCC. Shown are the boxplots of the abundance of B cells, cancer-associated fibroblasts, macrophage, and endothelial cells in different clusters of type Ⅱ pRCC.

Alt text: Boxplots showing the distribution of several immune cell types across different type Ⅱ pRCC clusters.

To assess the prognostic value of the 10 hub genes, all patients were divided into two groups based on the median expression value of hub genes: those with expression levels higher than or equal to the median value were classified as the high-level group, while those below the median were assigned to the low-level group. Kaplan-Meier analysis showed that five hub genes (i.e., *RRM2, ADCY3, CCNA2, FLNA*, and *CENPF*) were significantly associated with prognosis (*P*<0.05). Higher expression levels of these five hub genes were correlated with poorer prognosis in type II pRCC patients (See Figure S6). Among these, *RRM2*, a gene within the p53 signaling pathway, is a known maker of poor prognostic in pRCC patients [20]. Compared with pRCC patients with high *RRM2* expression, patients with low *RRM2* expression have better clinical outcomes. Similarly, elevated *CENPF* expression in advanced pRCC is associated with poor survival outcomes and increased mortality [21]. The other three hub genes, *ADCY3*, *CCNA2*, and *FLNA*, were identified as key genes associated with the survival and prognosis of type Ⅱ pRCC, which have not been previously reported or confirmed. We hope our findings will guide future research in exploring molecular targets for type II pRCC.


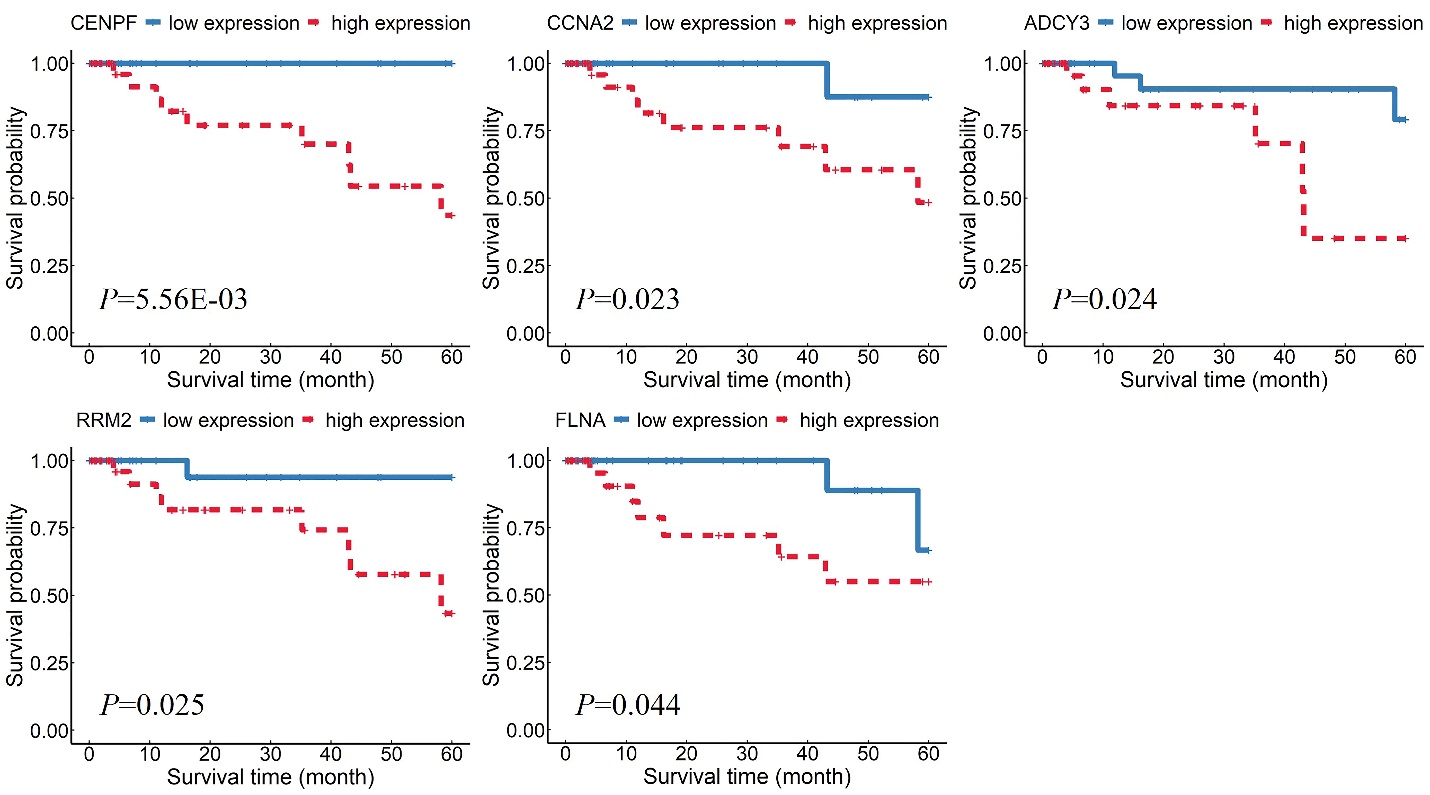


**Figure S6.** Plots of prognostic survival curves of the 5 hub genes in type Ⅱ pRCC sorted in ascending order by *P*-values.

Alt text: Kaplan–Meier survival plots showing prognostic curves for nine hub genes in type Ⅱ pRCC.

**9. Supplementary Note 9: Real data analysis on LGG** **and BRCA**

We also applied iMKL to molecular subtyping of multi-omics data in Low-Grade Gliomas (LGGs) and Breast Invasive Carcinoma (BRCA) from The Cancer Genome Atlas Program (TCGA). We considered three omics data types of each cancer, namely miRNA expression, mRNA expression, promoter CpG methylation from TCGA. After applying data preprocessing procedures, the data used for analysis contained 17,034 mRNAs, 501 miRNAs, and 16,276 methylation features for the 499 LGG patients and 8,404 mRNAs, 404 miRNAs, and 16,320 methylation features for 676 BRCA patients from TCGA.

The results indicate that 499 LGG patients and 676 BRCA patients were each divided into two subtypes with significant different prognoses ($P$-value =1.56E-14 for LGGs, $P$-value =1.68E-03 for BRCA). The corresponding survival curves are illustrated in Figure S7, which indicates that iMKL also demonstrates good performance and robustness in identifying LGG and BRCA molecular subtypes with significant differences and provides a broader perspective on the model's applicability.


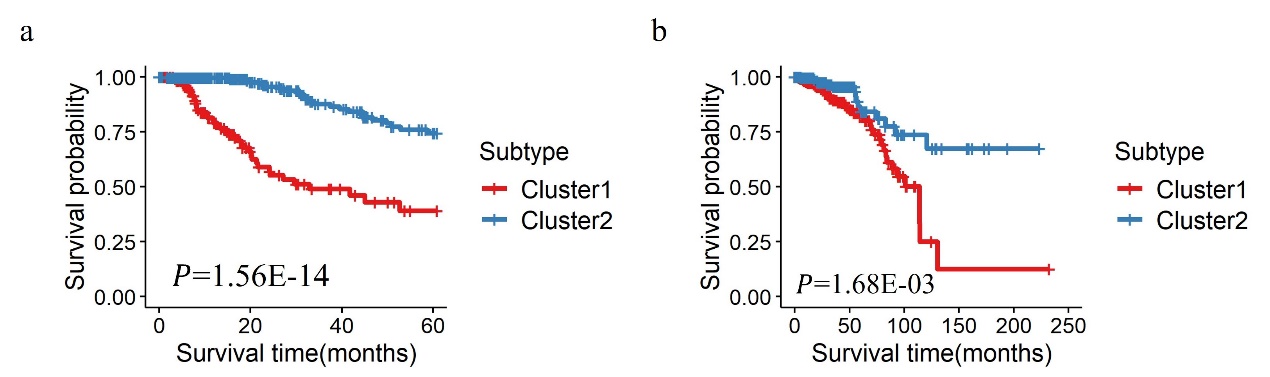


Figure S7. Subtyping results of 499 LGG patients (a) and 676 BRCA patients (b) using iMKL.

Alt text: Kaplan-Meier survival curves showing the subtyping results obtained by iMKL for LGG patients and BRCA patients.

**Reference**

1. Bray F, Laversanne M, Sung H, et al. Global cancer statistics 2022: GLOBOCAN estimates of incidence and mortality worldwide for 36 cancers in 185 countries, CA: A Cancer Journal for Clinicians 2024;74:229-263.

2. Jiang A, Li J, He Z, et al. Renal cancer: signaling pathways and advances in targeted therapies, MedComm 2024;5(8):e676.

3. Linehan WM, Ricketts CJ. The Cancer Genome Atlas of renal cell carcinoma: findings and clinical implications, Nat Rev Urol 2019;16:539-552.

4. Cancer Genome Atlas Research N, Linehan WM, Spellman PT, et al. Comprehensive Molecular Characterization of Papillary Renal-Cell Carcinoma, N Engl J Med 2016;374:135-145.

5. Colaprico A, Silva TC, Olsen C, et al. TCGAbiolinks: an R/Bioconductor package for integrative analysis of TCGA data, Nucleic Acids Res 2016;44:e71.

6. Gusev A, Lee SH, Trynka G, et al. Partitioning heritability of regulatory and cell-type-specific variants across 11 common diseases, Am J Hum Genet 2014;95:535-552.

7. Troyanskaya O, Cantor M, Sherlock G, et al. Missing value estimation methods for DNA microarrays, Bioinformatics 2001;17:520-525.

8. Rousseeuw PJ. Silhouettes: A graphical aid to the interpretation and validation of cluster analysis. Journal of Computational and Applied Mathematics 1987;20:53-65.

9. Wijaya Y A, Kurniady D A, Setyanto E, et al. Davies bouldin index algorithm for optimizing clustering case studies mapping school facilities. TEM J, 2021;10(3): 1099-1103.

10. Harrell FE, Lee KL, Mark DB (1996) Tutorial in biostatistics multivariable prognostic models: issues in developing models, evaluating assumptions and adequacy, and measuring and reducing errors. Stat Med 15:361–387.

11. Antolini L, Boracchi P, Biganzoli E. A time‐dependent discrimination index for survival data, Statistics in medicine 2005;24(24): 3927-3944.

12. Sun Z, Xiao B, Jha H C, et al. Kaposi's sarcoma-associated herpesvirus-encoded LANA can induce chromosomal instability through targeted degradation of the mitotic checkpoint kinase Bub1, Journal of virology 2014;88(13): 7367-7378.

13. Xi Q, Huang M, Wang Y, et al. The expression of CDK1 is associated with proliferation and can be a prognostic factor in epithelial ovarian cancer, Tumor Biology 2015;36(7): 4939-4948.

14. Kankaya D, Kiremitci S, Tulunay O, et al. Gelsolin, NF-kappaB, and p53 expression in clear cell renal cell carcinoma: Impact on outcome, Pathol Res Pract 2015;211:505-512.

15. Rathmell W K, Rathmell J C, Linehan W M. Metabolic pathways in kidney cancer: current therapies and future directions, Journal of Clinical Oncology 2018:36(36): 3540-3546.

16. Broad RV, Jones SJ, Teske MC, et al. Inhibition of interferon-signalling halts cancer-associated fibroblast-dependent protection of breast cancer cells from chemotherapy, Br J Cancer 2021;124:1110-1120.

17. Steger J, Cole AG, Denner A, et al. Single-cell transcriptomics identifies conserved regulators of neuroglandular lineages, Cell Rep 2022;40:111370.

18. Zhang F, Liu L, Wu P, et al. Overexpression of MAX dimerization protein 3 (MXD3) predicts poor prognosis in clear cell renal cell carcinoma, Transl Androl Urol 2021;10:785-796.

19. Martinez Chanza N, Xie W, Asim Bilen M, et al. Cabozantinib in advanced non-clear-cell renal cell carcinoma: a multicentre, retrospective, cohort study, Lancet Oncol 2019;20:581-590.

20. Wang H, Wang X, Xu L, et al. A pan-cancer perspective analysis reveals the opposite prognostic significance of CD133 in lower grade glioma and papillary renal cell carcinoma, Sci Prog 2021;104:368504211010938.

21. Trevisani F, Floris M, Vago R, et al. Long Non-Coding RNAs as Novel Biomarkers in the Clinical Management of Papillary Renal Cell Carcinoma Patients: A Promise or a Pledge?, Cells 2022;11(10):1658.

1. *Corresponding author: [caohy@sxmu.edu.cn](mailto:caohy@sxmu.edu.cn) (H. Cao), [cuiy@msu.edu](mailto:cuiy@msu.edu) (Y. Cui) [↑](#footnote-ref-1)
